# Epimorphin Regulates Bile Duct Formation via Effects on Mitosis Orientation in Rat Liver Epithelial Stem-Like Cells

**DOI:** 10.1371/journal.pone.0009732

**Published:** 2010-03-17

**Authors:** Junnian Zhou, Lei Zhao, Lipeng Qin, Jing Wang, Yali Jia, Hailei Yao, Chen Sang, Qinghua Hu, Shuangshuang Shi, Xue Nan, Wen Yue, Fengyuan Zhuang, Chun Yang, Yunfang Wang, Xuetao Pei

**Affiliations:** 1 Stem Cell and Regenerative Medicine Lab, Beijing Institute of Transfusion Medicine, Beijing, China; 2 School of Biological Science and Medical Engineering, Beihang University, Beijing, China; City of Hope National Medical Center, United States of America

## Abstract

Understanding how hepatic precursor cells can generate differentiated bile ducts is crucial for studies on epithelial morphogenesis and for development of cell therapies for hepatobiliary diseases. Epimorphin (EPM) is a key morphogen for duct morphogenesis in various epithelial organs. The role of EPM in bile duct formation (DF) from hepatic precursor cells, however, is not known. To address this issue, we used WB-F344 rat epithelial stem-like cells as model for bile duct formation. A micropattern and a uniaxial static stretch device was used to investigate the effects of EPM and stress fiber bundles on the mitosis orientation (MO) of WB cells. Immunohistochemistry of liver tissue sections demonstrated high EPM expression around bile ducts *in vivo*. *In vitro*, recombinant EPM selectively induced DF through upregulation of CK19 expression and suppression of HNF3α and HNF6, with no effects on other hepatocytic genes investigated. Our data provide evidence that EPM guides MO of WB-F344 cells via effects on stress fiber bundles and focal adhesion assembly, as supported by blockade EPM, β1 integrin, and F-actin assembly. These blockers can also inhibit EPM-induced DF. These results demonstrate a new biophysical action of EPM in bile duct formation, during which determination of MO plays a crucial role.

## Introduction

Duct formation (DF) is an important process in development and regeneration of many epithelial organs including lung, kidney, mammary glands and liver, and is known to be regulated by diffusible morphogens and elements of the insoluble extracellular matrix [1]. In addition to the biochemical signaling pathways (such as MAPK and FAK signaling) related to regulating the expression of various marker genes, information about biophysical properties is also crucial to DF. Intrahepatic bile ducts, a series of tubules transporting bile produced by hepatocytes to the gallbladder, are an important duct system within the liver. The lumen of these bile ducts is lined with biliary epithelial cells which share a common origin with hepatocytes [2]. The close association between biliary epithelial cells and the basement membrane leads to a hypothesis that extracellular matrix components of the portal mesenchyme are important in controlling biliary epithelial cell differentiation via cell-matrix interactions [2]. The mechanism for controlling bile duct formation, especially the effect of biophysical properties, however, remains largely unknown. While it is known that extracellular matrix complexes such as Matrigel can combine with soluble growth factors to meet the minimum requirements for DF of hepatoblasts *in vitro* [3], little is known about the function of single matrix proteins in DF.

Epimorphin (also known as syntaxin 2), a mesenchymal cell-associated membrane protein, functions as a key epithelial morphoregulator in various organs including lung, mammary gland, pancreas, gallbladder, intestine, and sex glands [4], [5]. One key event in EPM-directed morphogenesis is epithelial DF. In liver, EPM, expressed on hepatic stellate cells specifically [6], is reported to be involved in liver regeneration [7]–[9] and morphogenesis [10]–[12], however, little is known about the role of EPM in bile duct formation. This may be due to the low percentage of biliary epithelial cells in the liver or a lack of suitable model for cell differentiation into biliary epithelial cells *in vitro*. Herein, we focused on the effects of EPM on bile duct formation, a typical epithelial DF in liver morphogenesis. *In vitro* experiments demonstrated that depending on the context of protein presentation, EPM can selectively direct two key processes of tubulogenesis: branching morphogenesis (involved in tubule initiation and extension) and luminal morphogenesis (required for the tubule caliber enlargement) in mammary cell clusters [13]. It was proposed that EPM presentation and topological orientation might in turn control mitotic spindle axis orientation [4]. No direct experimental evidence, however, has been presented on EPM regulation of MO or MO's involvement in tubulogenesis.

WB-F344 cells, a well characterized culture model system of bipotential hepatic precursor cells (for review see ref. [14]), are capable of hepatic [15], [16] and biliary differentiation [17], [18], [19] both *in vitro* and *in vivo*, with differentiation towards biliary duct cells occurring when the cells are seeded onto Matrigel *in vitro* [18], [19]. It is worth noting that EPM can be found in preparations of Matrigel [20]. Considering that EPM but not HGF or EGF is well established as one of the primary morphogens in tubulogenesis [4], [5], we therefore hypothesized that EPM may be a factor involved in formation of duct-like structures by WB cells through guidance of MO in a biophysical pathway.

In the present study, we aimed to shed light on the biophysical role of recombinant EPM in biliary duct formation. A micro-pattern fabrication device and a static uniaxial stretch system were developed to study the biophysical mechanism of EPM.

## Results

### EPM Is Located Close to the Newly Generated or Normal Bile Ducts *In Vivo*

**Figure 1:**
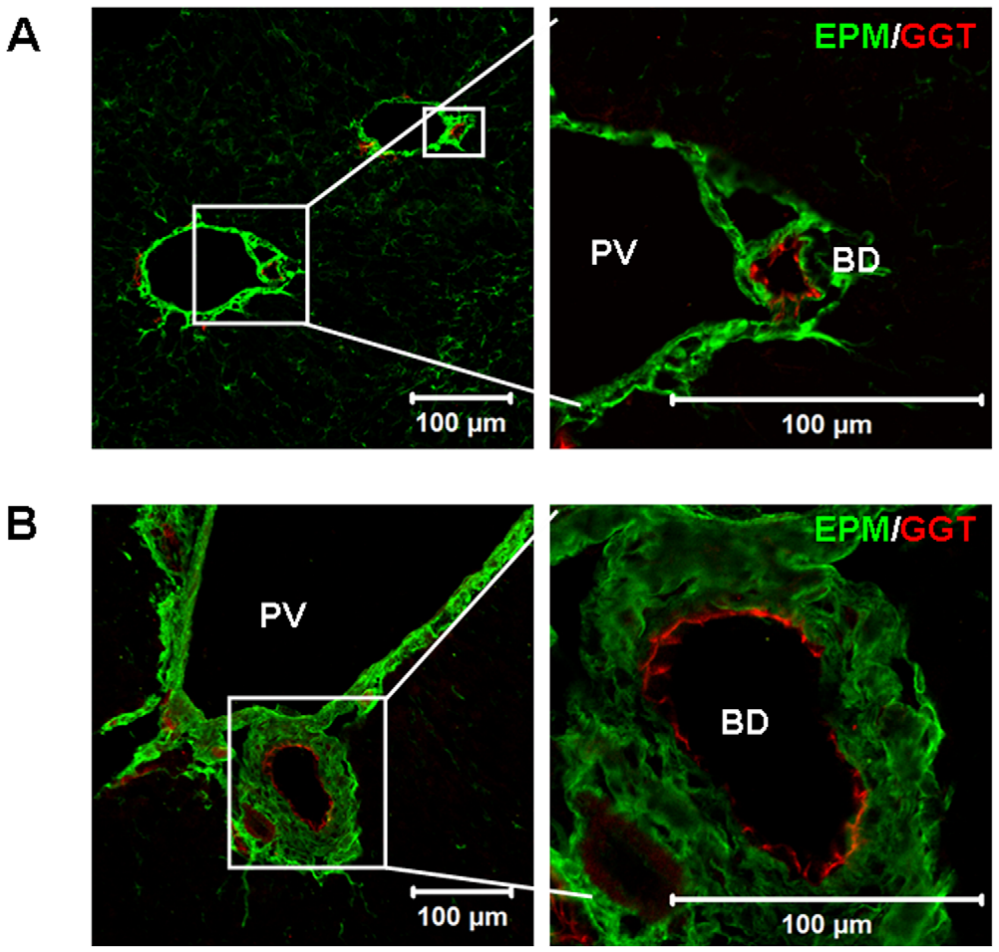
Dual immunofluorescence for EPM and GGT in liver frozen sections. Adult male C57Bl/6 mice were given a single intraperitoneal 10% solution in corn oil injection of CCl_4_ (10 µl/g body weight) or the same volume corn oil as a control. The CCl_4_-treated mice (A) or normal control mice (B) were sacrificed to obtain liver tissues at day-7, which were cut into 8 µm frozen liver sections. Biliary epithelial cells were specifically stained with GGT (red). EPM (green) was strongly expressed in the mesenchyme surrounding GGT-positive BD. The diameter of the BD: (A) 11.52–14.04 µm; (B) 33.38–80.24 µm. PV  =  portal vein. BD  =  bile ducts. Pictures on the right are magnifications of pictures on the left. The BD was shown in the square region. The diameter of the BD was measured by Image-Pro Plus 6.0 software (Media Cybernetics, MD, USA). Bars  = 100 µm.

Previous studies clearly demonstrated the common expression of EPM in connective tissue around epithelial cells of various organs [21]. EPM was reported to be expressed in liver in areas along the sinusoidal lining and in the connective tissues around blood vessels [21]. EPM expression can be enhanced around portal veins after acute liver injury such as partial hepatectomy [9] or after injection of CCl_4_ [7], [8]. In this study, we found a strong and previously undocumented expression of EPM in the mesenchyme around the bile ducts in the liver of CCl_4_-treated (**Fig. 1A**) and normal adult mice (**Fig. 1B**). The negative control is shown in **Fig. S1**. Consistent with the previous reports [22], some new regenerating small bile ducts were observed in the liver of CCl_4_-treated mice (**Fig. 1A**). Figure 1 suggested to us a close association of EPM with bile ducts. Our results may also extend the previous observations that bile ducts, hepatic blood vessels and mesenchyme development is cross-regulated [2] since EPM is highly expressed on mesenchyme around both bile ducts and blood vessels. These results suggest that EPM may be involved in maintaining normal morphogenesis and/or regenerating bile ducts *in vivo*, and prompted us to investigate whether EPM plays a role in the duct-like differentiation of WB-F344 cells into biliary lineage.

### EPM Induces Bile Duct-like Structures of WB-F344 Cells *In Vitro*

**Figure 2:**
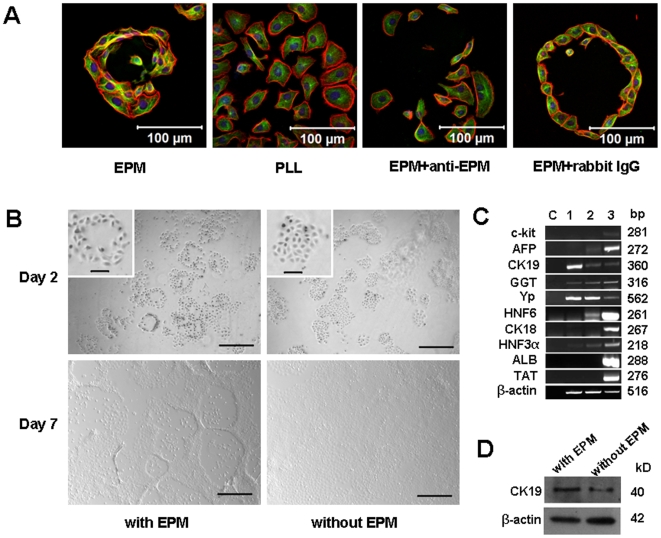
The morphogenesis and hepatobiliary genes analysis of the WB cells treated with recombinant EPM. (A) Confocal images of EPM-induced duct-like structures. The PLL was used as a matrix control, coated with EPM or alone. The duct-like structures appeared 24 hours to 48 hours after cell seeding. The formation of these duct-like structures could be blocked by the EPM-neutralizing antibody with the purified rabbit IgG as a control. Bars  = 100 µm. Tubulin, green. F-actin, red. Nuclei, blue. (B) Brightfield images of EPM-induced WB cells. The typical duct-like structures appeared at day-2. The inset shows a magnified view of a duct-like structure and normal WB cells. Individual duct-like structures developed into cord-like structures at day-7 while the WB cells cultured on EPM-free surface remained polygonal and small. Bars  = 500 µm (Bars in inset  = 100 µm).(C) RT-PCR analysis of WB cells treated with or without EPM at day-7. Lane C: water was substituted for the template as a negative control. Lane 1, cultured on EPM-coated dishes. Lane 2, cultured on EPM-free dishes. Lane3, adult rat liver (liver samples of AFP were from E17.5 rats). The 25 cycle β-actin bands were used as an internal control for semi quantitative RT-PCR. (D) Western blot analysis of the expression of 40-kDa CK19. 42-kDa β-actin was used as an internal control. An increased expression level of CK19 was detected in the EPM-treated cells.

In our study, WB-F344 cells were cultured on EPM-coated substrate in the presence of 5% FBS-containing medium without any growth factors. At low cell density condition (3×10^3^ cells/cm^2^), individual duct-like structures derived from single colonies appeared only on EPM-coated but not on poly-L-lysine (PLL)-coated substrata 24 to 48 hours after cell seeding (**Fig. 2A**). These 2-D structures were similar to the bile ducts observed in liver sections (**Fig. 1**). Importantly, formation of these duct-like structures could be blocked by the EPM-neutralizing antibody, while duct-like structures were still retained in the representative rabbit IgG control group (**Fig. 2A**). These phenomena indicate an important and specific effect of EPM as a matrix protein on inducing DF in WB-F344 cells.

Individual duct-like structures developed into cord-like structures at day-7, while WB-F344 cells cultured on the EPM-free surface were still polygonal and small. Empty spaces of the cords were gradually colonized by polygonal cells (**Fig. 2B**), a morphological change similar to that of the cells cultured on Matrigel reported by Couchie et al. [18]. EPM also had a slight inhibitory effect on WB-F344 cell proliferation as reported previously (**Fig. S2**) [11], [13], [23].

We subsequently analyzed hepatobiliary gene expression of EPM-treated WB-F344 cells at day-7 at the mRNA or protein levels. In the absence of EPM, WB-F344 cells tested negative for c-kit, albumin (ALB), CK18, and tyrosine aminotransferase (TAT) and tested positive for α-fetoprotein (AFP), CK19, gamma glutamyl transpeptidase (GGT), and glutathione-S-transferase pi (Yp). This profile matches the profile reported for hepatoblasts [14], [24]. Interestingly, in EPM-treated WB-F344 cells, hepatocytic lineage genes, including ALB, CK18, and TAT, were not induced except for the up-regulation of CK19 (**Fig. 2C,D**), a common bile duct marker in mammalian tissues [25]. Functional biliary gene expression levels were not changed. Genes associated with hepatoblasts and/or hepatocytes, including AFP, and hepatocyte nuclear factors (HNF) 3α and 6, were suppressed (**Fig. 2B**). HNF3α is a transcription factor known to regulate hepatocyte-specific genes in liver [26]. HNF6 is reported to promote hepatocyte over biliary differentiation by attenuating early biliary-cell commitment [27], [28]. These results suggest that the duct-like structures induced by EPM in our system are biliary rather than hepatocytic. Although the liver and pancreas share a common origin in development, the trans-differentiation of WB-F344 cells into pancreatic endocrine cells has not been achieved except when transfecting the PDX-1 gene as reported [29]. Thus, these CK19 upregulated ducts are mostly immature biliary ducts instead of pancreatic ducts in our system.

In the present study, our results in WB-F344 cells are different from those found by Miura et al., in which EPM seemed to have the same effect on biliary and hepatocytic differentiation that rat primary hepatic stem-like cells did [11]. Our data suggests that EPM has a specific effect on bile duct-like formation rather than hepatocyte-like differentiation. This difference may be due to the heterogenicity of primarily isolated hepatic stem-like cells rather than the clonal WB-F344 cell line used in this study.

### EPM Guides the Orientation of WB-F344 Cells during Mitosis

**Figure 3:**
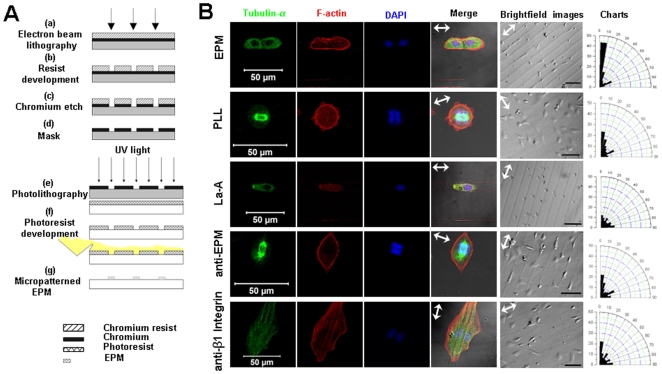
Microfabrication of lines and the effects of patterned EPM on WB cell orientation during mitosis. (A) A schematic of the method used to fabricate EPM protein lines on coverslips. Using standard photolithography methods, microarrays of parallel lines (10 µm width, 40 µm spacing)) were manufactured on borosilicate glass wafers. The photoresist was spun onto a borosilicate wafer and exposed to UV light through an optical mask containing the desired pattern to degrade the photoresist (a–f). EPM solution or a mixture of EPM and PLL (for the blocking experiment) was deposited on the patterns (5 µg/cm^2^) which were dried at room temperature and then stored overnight. The remaining photoresist was dissolved in acetone (g). Bovine serum albumin and PLL on the same micropatterned substrate were used as control. (B) Observations and analyses of direction of division of cells grown on the EPM lines 24 hours after seeding. Before seeding on the micropattern, WB cell division was synchronized using a double-thymidine block at 2 mM. Only the individual growing cells at metaphase and telophase were counted, while cell clusters were excluded from counting. The MO of the cells was determined by the line of nucleus centers or the perpendicular line of the equatorial plate. Dividing cells were visualized by confocal laser scanning microscope. The arrowheads in the merged images and brightfield images indicated EPM line directions. Brightfield images illuminated the cells grown on the micropatterns. These cells were divided into 9 groups (a 10°-wide sector for each group) according to the angles between the MO and the line directions. The polar coordinate charts showed that the MO of the cells on EPM (cell number: n = 192) was predominantly in the direction of the line in contrast to a rather random distribution of MO of the cells on the PLL (cell number: n = 82) patterns. The MO of the La-A-treated cells on EPM patterns was measured by phase-contrast images in which metaphase plates were easily visible. Cells treated by La-A (cell number: n = 83), EPM-neutralizing antibody (cell number: n = 68), and β1 integrin-neutralizing antibody (cell number: n = 54), all demonstrated a rather random distribution in their MO. Bars, brightfield images: 100 μm, confocal images: 50 μm.

*In vivo*, the bile ducts are formed not through branching but through lumen morphogenesis by the remodeling of the ductal plates in contrast to the morphogenesis that occurs in the lung and mammary gland. Since MO determination has been proposed as crucial to the two distinct processes of EPM-induced mammary epithelial morphogenesis [4], we investigated the role of EPM on MO determination in WB-F344 cells by a microfabrication technique (**Fig. 3A**). Microfabrication provides a powerful approach for studying cell-matrix interactions in regulating cell function [30], [31]. Cells spread on the protein lines were all fibroblast-like within 24 hours after seeding with their long axes preferentially parallel to the pattern lines.

Some cells grown on EPM lines had division orientations aligning along the direction of the lines (43% in a 10°-wide sector). The rest (57%) were more evenly distributed in an 80°-wide sector (**Fig. 3B**). Cells treated with EPM-neutralizing antibodies demonstrated a rather random distribution in their MO, which was similar to the PLL (**Fig. 3B**) or BSA (**Fig. S3**) controls. These results suggest that EPM has the ability to regulate MO determination of WB-F344 cells.

Noting that the cells plated on EPM-coated lines actually adopt the shape imposed by the lines, one could easily embrace the hypothesis presented by Oscar Hertwig [32] in the 19^th^ century, in which it was claimed that regulation of cell shape may contribute to MO determination. However, later research by Théry et al. [33], [34] demonstrated that spindle orientation is not solely driven by cell shape but rather by the spatial distribution of the extracellular matrix proteins and cortical cues. Recently, both integrin signaling [35]-[37] and stress fibers (i.e. F-actin) [38] were reported to be essential to MO determination. As heterodimeric transmembrane receptors, integrins connect the extracellular matrix to the cell's stress fibers [39], [40]. Thus, stress fiber assembly and EPM receptor (β1 integrin) blocking experiments were carried out to explore their roles in causing EPM to affect MO. For the latrunculin A (La-A) and β1 integrin-neutralizing antibody-treated cells, division directions became randomly oriented (**Fig. 3B**), which was similar to a recent report [35]. These results indicate that β1 integrin and stress fibers are key mediators in EPM-induced MO determination.

### Determination of Mitosis Orientation Is Secondary to Stress Fiber Orientation

In addition to microtubules, stress fibers have recently been implicated in mitotic spindle positioning [38]. Our La-A treatment results in micropattern experiments also indicate a potential role of stress fibers in EPM-guiding MO determination. To confirm the effects of stress fiber orientation (SFO) on guiding MO in an exogenous biochemical factor-free environment, we established a modified static-uniaxial-stretch system, a way to compel SFO parallel with the direction of static uniaxial stress (**Fig. S4A, B**) [41]. In this study, an 8% strain was exerted on adherent cells for 24 h. SFO was then found to be preferentially parallel to the static strain direction (**Fig. S4C,D**). In the stretched cells, SFO and MO aligned mainly along the stress direction (24% and 26% of the cells were in the first 10°-wide sector, respectively) (**Fig. S4D**). Among the cells of which SFO aligned in the first 30°-wide sector, 87% of their MO was also in the first 30°-wide sector (**Table S1**). These results suggest a high correlation between the SFO and MO.

To further confirm that MO is secondary to SFO, La-A was used to depolymerize the F-actin bundles in the stretched cells. La-A-treated cells exhibited random cell division alignment, which diminished the correlation between MO and the strain (**Fig. S4C,D**).

These results from our micropattern and static-uniaxial-stretch experiments strongly implicate EPM in guiding the MO of WB-F344 cells along the direction of EPM pattern lines in which β1 integrin and stress fiber alignment is involved.

### Focal Adhesion Assembly, Stress Fibers Alignment and Subsequent MO Determination Are All Required in EPM-induced Bile Duct Formation

The data described above suggests that EPM has two features in the WB-F344 cell model: (i) induction of WB-F344 cells into biliary ducts and (ii) guidance of MO determination mediated by β1 integrin and stress fibers. In order to elucidate the mechanism of EPM's effect on DF, we analyzed the stress fiber alignment, focal adhesion assembly, and MO determination of EPM-treated WB-F344 cells during differentiation and their effects on DF.

**Figure 4:**
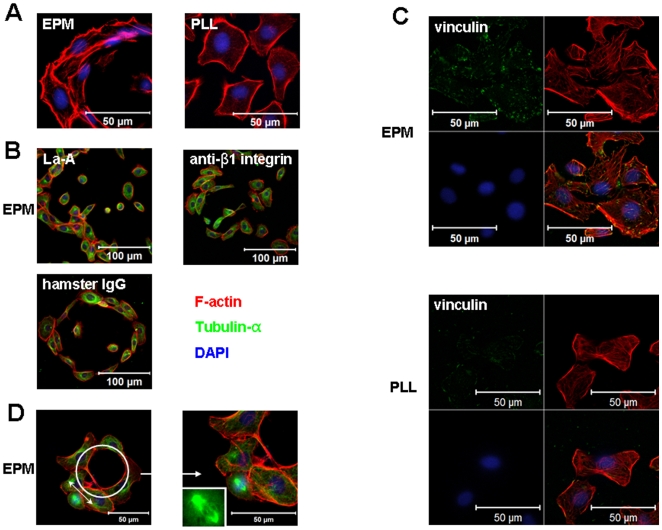
Effects of F-actin, β1 integrin, focal adhesions and MO on duct formation. (A) Confocal images of stress fiber (F-actin) alignment of WB cells treated with EPM and PLL. Bars  = 50 µm. (B) For inhibition experiments, the duct-like structures that formed were treated with La-A at 0.5 µM, which can disrupt microfilament polymerization, for 2 hours before the cells were fixed. For β1 integrin-neutralizing experiments, the WB cells had a pre-incubation with anti-β1 integrin antibody or armenian hamster IgG for 30 minutes at 37°C before they were seeded with antibodies together. Bars  = 100 µm. (C) Confocal images of focal adhesions of the WB cells. Focal adhesions were visualized by vinculin (green) 4 hours after cell seeding. Bars  = 50 µm. (D) A typical WB cell in division in a duct-like structure induced by EPM. The image in the right is a magnification of the image on the left. The inset shows a magnified view of the spindle. Bars  = 50 µm.

First, we noted that F-actin microfilament bundles (i.e. stress fibers) appeared to be randomly oriented in cells on EPM-free substrata, while in cells grown on EPM substrata, actin cables were organized into cortical bundles predominantly arranged along the long axes of the cells (**Fig. 4A**). To ascertain whether stress fibers are associated with DF, La-A was added to the EPM induction system. La-A-treated cells, mostly round in shape and smaller than normal cells, were gradually separated from the duct-like structures (**Fig. 4B**).

Extra attention was paid to focal adhesions because they connect the extracellular matrix to stress fibers in the cytoplasm via integrin. We first stained vinculin, a component of focal adhesions, in order to visualize these focal adhesions. Our results showed that EPM promoted the assembly of focal adhesions compared with the effect of PLL within 4 hours (**Fig. 4C**). This is consistent with the previous cell adhesion assays [13] and the fact that EPM is bound to αvβ1 integrin [42], a focal adhesion assembly protein. We then found that β1 integrin-neutralizing antibody as well as the EPM-neutralizing antibody could also disrupt the EPM-induced DF of the WB-F344 cells, while WB-F344 cells still retained representative duct-like structures in the hamster control IgG samples (**Fig. 4B**). We also observed dividing cells along the tangent direction of the duct-like structure which extended the micropattern results from 1-D to 2-D (**Fig. 4D**).

Taken together, the factors investigated (β1 integrin- and EPM-neutralizing antibodies, and La-A) which could disrupt MO determination in our experiments suppressed the formation of bile duct-like structures by WB-F344 cells.

## Discussion

Understanding how hepatic precursor cells differentiate into bile ducts is crucial for epithelial morphogenesis studies and for the development of future therapies for hepatobiliary diseases. Hepatic precursor cell induction and differentiation could be achieved by adding specific growth factors or inhibitors of signaling pathway in combination with coating the extracellular matrix proteins in an *in vitro* culture system. As a histone deacetylase inhibitor, sodium butyrate can increase CK19 and GGT expression in WB-F344 cells on a plastic surface [18], however, it failed to induce the duct-like structures of WB-F344 cells without coating EPM-contained Matrigel [18], [19] or EPM alone in our study. Recently, matrix matalloproteinase (MMPs) were identified as effectors downstream of EPM-induced epithelial morphogenesis [10], [43], but MMPs do not work in inducing duct morphogenesis of the epithelial cell line in the absence of EPM *in vitro* [43]. These limitations suggest that biochemical pathways alone are not sufficient to induce DF. Our data indicates that EPM is involved in the duct morphogenesis of hepatic stem-like cells (WB-F344 cells) via a putative biophysical mechanism.

**Figure 5:**
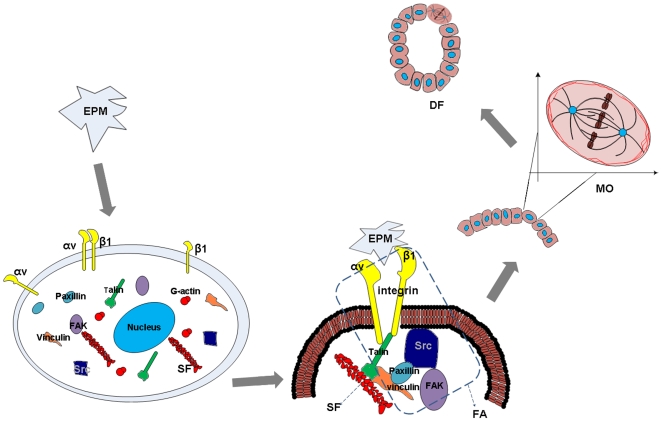
Hypothetical model for mechanisms of action of EPM on duct formation by WB cells *in vitro*. ***EPM***: Epimorphin. Unlike the control groups on PLL, cell contact with EPM showed a rapid induction of focal adhesions of which αvβ1 integrin, the EPM receptor, is one of the components. Our results showed that EPM had an effect on stress fiber alignment, MO and bile duct-like structure formation, when compared with the PLL control. Proof of the effects on MO and DF were given in our EPM neutralizing experiments. ***FA***: Focal adhesion. Stress fibers in the cytoplasm are connected to the β1 integrin-containing FA on the cellular membrane. Our data and those of others suggest that blockade of αvβ1 integrin results in disoriented spindles [35] and a loss of key processes in cyst formation [42]. FAK and Src, as components of focal adhesion, are downstream molecules in the EPM biochemical signaling pathway [42]. ***SF***: Stress fiber. The guidance of MO by a patterned EPM protein can be blocked by La-A, which suggests an important role of SFO in guiding the mitosis orientation of WB cells. The fact that the high correlation between MO and the uniaxial static stretch direction can be blocked by La-A provides additional evidence that the SFO determines MO. Disassembly of stress fibers can also block the duct formation induced by EPM. ***MO***: Mitosis orientation. Radisky et al. proposed that EPM might be involved in duct formation by guiding MO [4]. The present study shows that all MO blockers used in our experiments (EPM neutralizing antibody, β1 integrin neutralizing antibody, and La-A) can also suppress the bile duct-like structure formation induced by EPM. ***DF***: Duct formation. *In vivo* results demonstrated that EPM is involved in bile duct formation in CCl_4_-injured or healthy adult mice (**Fig. 1**). Our analysis indicates that these ducts are biliary ducts rather than hepatocytic ducts (**Fig. 2**).

In the present work, we first demonstrated that EPM is expressed around liver bile ducts and is involved in the MO determination and DF of WB-F344 cells. Based on our data, and reports by others [4], [34], we therefore proposed a putative biophysical morphogenic pathway for EPM, one of the primary proteins for duct morphogenesis (**Fig. 5**). EPM has the ability to guide MO determination of WB-F344 cells along the tangential direction of a cell-EPM contact surface via mediating focal adhesion assembly and F-actin bundle alignment, which may be vital to the bile duct-like formation of WB-F344 cells. We provided experimental support for this hypothesis by the following: (a) EPM exhibited effects on focal adhesion assembly, stress fiber alignment, MO determination and duct-like structure formation of WB-F344 cells compared with the PLL control. Proofs of the effects were given in the EPM-blocking experiments. (b) The blockage of β1 integrin, a component of focal adhesions which connect stress fibers and extracellular matrix, resulted in disoriented cell division directions and a loss of DF. (c) SFO played an important role in guiding the MO of WB-F344 cells, of which proof was given by our uniaxial stretch and blocking experiments. (d) All of the factors (β1-integrin-neutralizing antibody, EPM-neutralizing antibody, and La-A) disrupted MO determination but also suppressed the bile duct-like structure formation of WB-F344 cells.

Here, we revealed an interesting role of EPM in MO determination of WB cells. We wondered whether MO determination is essential or merely sufficient in the EPM-induced DF of WB cells. An elegant study by Théry et al. demonstrated that fibronectin has a role in determining the orientation of the division axis of HeLa cells [34], however, we found that fibronectin did not induce DF of the WB cells in our system (**Fig. S5**). Therefore, EPM is likely to be more specific than fibronectin in duct morphogenesis. This phenomenon suggests a significant role of EPM's downstream biochemical pathways of EPM beyond its immediate biophysical effect. In our experiments, since EPM had an enhancing effect on expression of CK19 and a reducing effect of HNF3α and HNF6 levels, we concluded that spatial distribution of EPM (a biophysical property) and its downstream signals (biochemical events) are both required for duct morphogenesis.

Noting that some other functional biliary genes such as GGT and Yp were not elevated, we presumed that EPM may play an earlier and conserved role in the process of bile duct formation from WB-F344 cells, while other growth factors may also be required for the later stages of bile duct maturation. Bile duct morphogenesis, a process orchestrated by hepatic stem/progenitor cells and adult epithelial or mesenchymal cells, is governed by cell-matrix contact, by cell-cell contact, and by soluble growth factors [2]. As one of the various possible regulators reported for duct morphogenesis including laminin, fibronectin, collagen, HGF, and EGF [2], EPM is not likely to be responsible for this process alone. How these factors and their subsequent pathways are intertwined in the differentiation of WB-F344 cells into biliary lineage is worthy of future inquiry. As cell adhesion receptors for EPM and other extracellular matrix proteins including fibronectin, laminin, collagen, the roles of integrins such as cell fate determination [44], mitotic machinery regulation [44], [45], in hepatic stem/progenitor cell differentiation and/or morphogenesis, should also be paid more attention in further studies.

## Materials and Methods

### Animal Ethics Statement

The C57Bl/6 mice used in our experiments were purchased from the Laboratory Animal Center, Academy of Military Medical Science (Beijing, China) and were housed in the same facility according to the guidelines for laboratory animals treatment approved by the Beijing Experimental Animal Management Center.

### Cell Cultures, Synchronization, and Induction

Rat liver epithelial (stem-like) WB-F344 cells were derived from a normal adult male Fischer F344 rat as described previously [46], and has proven to be a useful model for studies of liver regeneration [47]. The WB-F344 cells were kindly provided by Dr. Zhongxiang Lin (Beijing Institute for Cancer Research, Beijing, China) and were maintained in RPMI 1640 (GIBCO, Grand Island, NY, USA), supplemented with 10% fetal bovine serum (FBS, Hyclone, Logan, UTA, USA), 200 IU/ml penicillin and 50 mg/ml streptomycin (GIBCO) at 5% CO_2_ and 37°C. For micropattern and uniaxial stretch experiments, the WB-F344 cells were synchronized in division using a double-thymidine block. Following a 12 hour thymidine (Sigma, St. Louis, Missouri, USA) block at 2 mM, the cells were released into normal medium for another 12 hours. This procedure was carried out twice.

Recombinant EPM protein (H123, 1-264aa, a generous gift from Dr. Hirai [13], [48]) and PLL (Sigma) were coated on coverslips or micropatterned slips at a concentration of 5 µg/cm^2^ alone or mixed together in EPM blocking experiment. Slips were dried for 2 hours at room temperature before seeding WB-F344 cells, at a density of 3.0×10^3^ cells/cm^2^ onto coated cover slips, micropattern slips, or 6-well plates (Corning, Corning, NY, USA) in RPMI 1640 medium containing 5% FBS (Hyclone).

To study the effects of EPM on WB-F344 cell growth, 1×10^3^ WB-F344 cells were seeded per well of the EPM-coated 96-well plates (5 µg/cm^2^). Cell numbers were determined daily using a Cell Counting Kit-8 (Dojindo Laboratories, Kamimashiki-gun, Kumamoto, Japan).

### Functional Blockage and Inhibition Experiments

For our blocking experiment, EPM neutralizing antibody (MC-1, 20 µg/ml, a generous gift from Dr. Hirai [13], [20], [49]) and LEAF™ purified β1 integrin neutralizing antibody (50 µg/ml, BioLegend, San Diego, CA, USA) were used, with rabbit IgG (Santa Cruz Biotechnology) and armenian hamster IgG (BioLegend) as controls, respectively. For the β1 integrin blocking experiment, the cells were pre-incubated with the antibody or control IgG for 30 minutes at 37°C before cell seeding. For inhibition experiments, the duct-like structures that formed were treated with La-A, which can disrupt microfilament polymerization, for 2 hours before the cells are fixed.

### Cells Staining

To stain the cytoskeletal proteins, treated WB-F344 cells were fixed in 4% paraformaldehyde (Sigma) for 20 minutes and permeabilized in 0.1% Triton X-100 for 15 minutes. After two washes in PBS and blocking with 5% goat serum and 1% BSA, the cells were incubated with mouse anti-tubulin-α antibody (Neomarkers, Fremont, CA, USA) diluted at 1∶100 in PBS containing 0.1% BSA at 4°C overnight followed by FITC-conjugated anti-mouse IgG (Jackson ImmunoResearch Laboratories, West Grove, PA, USA) and rhodamine-conjugated phalloidin (Invitrogen, Grand Island, NY, USA) diluted at 1∶50 for 30 minutes. To stain focal adhesions, mouse anti-vinculin antibody (Sigma) was used 4 hours after cell seeding on matrix coated surfaces. Nuclei were visualized by DAPI (Sigma). PBS plus 0.05% Tween 20 was used for the washes between each step.

### Dual Immunofluorescence of Liver Tissues

Male C57Bl/6 mice (7 weeks old, weight 18–25 g) were administered a 10% solution of CCl_4_ (Sigma) in corn oil intraperitoneally (10 µl per g body weight) or the same volume of corn oil as a control. The mice were euthanized 1 week after the initial treatment. Frozen liver tissues were cut into 8 µm sections using a HM 505E cryostat (Microm, part of Thermo Fisher, Walldorf, Gemany). Double immunostaining was performed for EPM and GGT. Liver sections were fixed in cold acetone for 10 minutes and blocked with 5% goat serum overnight at 4°C. The primary antibody combination consisting of the rabbit polyclonal antibody against GGT (1∶100, Santa Cruz Biotechnology, Santa Cruz, CA, USA) and the rat monoclonal antibody against mouse EPM (MC-1, 1∶100) was used. Primary antibodies were then visualized using FITC-conjugated goat anti-rat IgG and TRITC-conjugated goat anti-rabbit IgG (Jackson ImmunoResearch Laboratories). The diameter of the bile ducts was measured by Image-Pro Plus 6.0 software (Media Cybernetics, MD, USA).

### RT-PCR Analysis

Total RNA was extracted from cells induced for 7 days with or without EPM using Trizol reagent (Sigma) according to the manufacturer's instructions. Two micrograms of total RNA were reverse transcribed using AMV reverse transcriptase (TaKaRa Bio, Otsu, Shiga, Japan) to produce cDNAs. Amplification was performed in a GeneAmp 2700 thermal cycler (Applied Biosystems, Foster City, CA, USA). The PCR primer sequences used are listed in **Table 1**.

**Table 1:** Primers and conditions used for RT-PCR.

Gene name	Primer sequence (5′–3′)	Annealing temperature (°C)	Product length (bp)
AFP*	TGAAATTTGCCACGAGACGG TGTCATACTGAGCGGCTAAG	60	272
c-kit*	CATCATGGAAGATGACGAGC CAAATGTGTACACGCAGCTG	56	281
CK19*	TTGCGCGACAAGATTCTTGG CATCTCACTCAGGATCTTGG	60	360
GGT*	GTCACCAACTTCAACTCTGC CCTTATCACTGTTTACCTCGG	60	316
Yp	TGGAAGGAGGAGGTGGTTAC TGTCCCTTCGTCCACTACTG	55	562
HNF6	AGAACACGGGAAGGACAGA CAAGTGCTTGATGAAGACGA	53	261
CK18	CACCACCTTCTCCACCAACT TCGGTTTCCAGGTTCTTCAC	55	267
HNF3α*	TTCGGAGTTGAAGTCTCCAG CATATGCCTTGAAGTCCAGC	60	218
ALB*	GACAAGTTATGCGCCATTCC ACTGGGTCAGAACCTCATTG	60	288
TAT*	ACTCCTACGTGATTCAGACG TAACTTCAGGTTCTGTAGGC	60	276
β-actin	GTTGTCCCTGTATGCCTCTG GAGCCAGGGCAGTAATCTC	55	516

* [11]

### Western Blot Analysis

Protein extracts from WB-F344 cells contact with or without EPM for 7 days were isolated with standard RIPA buffer containing combinations of proteinase inhibitors including leupeptin, pepstatin A, aprotinin and PMSF (all from Sigma) overnight. Proteins were separated in 12% SDS–polyacrylamide gradient gels and subsequently blotted onto polyvinyllidene difluoride membranes (Millipore, Bedford, Massachusetts, USA) at 25 V for 30 minutes. Membranes were then washed with TBST, treated successively with 5% skim milk for 2 hours, and incubated with anti-CK19 antibody (1∶100, Santa Cruz Biotechnology) overnight at 4°C and HRP-labeled anti-goat antibodies (1∶1000, Jackson ImmunoResearch Laboratories) for an additional hour, with successive TBST washing for three times in each step. All blots were normalized with an antibody against β-actin (42 kDa, Jackson ImmunoResearch Laboratories) and protein was visualized with an enhanced chemiluminescence (ECL) labeling kit (Santa Cruz Biotechnology) according to the manufacturer's instructions.

### Micro-Pattern Fabrication

Microfabrication techniques were utilized to investigate the effect of EPM distribution on MO. In brief, electron beam lithography was carried out on a blank 5-inch chromium-on-glass optical mask coated with photoresist. After resist development, chromium etch was done in chrome-etchant 3144 Puranal (Honeywell International, Minneapolis, MN, USA). Optical mask fabrication was completed after resist dissolution in acetone (**Fig. 3Aa–d**) [50]–[54].

Using standard photolithography methods, microarrays of parallel lines (10 µm in width with 40 µm spacing) were manufactured on borosilicate glass wafers. The photoresist was spun onto a borosilicate wafer and exposed to UV light through an optical mask containing the desired pattern to degrade the photoresist (**Fig. 3A** **e**). After developing the photoresist, EPM solution (or a mixture of EPM and PLL from the EPM blocking experiment) was deposited on the pattern (5 µg/cm^2^) and stored overnight after drying at room temperature. The remaining photoresist was dissolved in acetone (**Fig. 3A** **g**). Pre-modified coverslips were soaked in 70% ethanol for sterilization and washed in PBS before cell seeding. Bovine serum albumin (Sigma) and PLL on the same micropatterned substrate were used as a control.

### Uniaxial Stretch Device

The uniaxial static stretch system was reconstructed from an equiaxial stretch system (**Fig. S4A**) [55]. A rectangular silicone strip (0.6 cm in width, F) was placed on the silicone membrane (D) which sealed the culture well. A uniaxial strain was produced in the strip by the displacement of the indenter (B). Before cell seeding, the strain in the strip was measured. Microcarrier beads were scattered on the strip. The displacement of the beads before and after the stretching was measured to calculate the strain (**Fig. S4B**).

### MO Observation and Statistic Analysis

Twenty-four hours after seeding, observations were carried out to analyze the direction of division of cells grown on EPM lines or cells exposed to uniaxial stretch. Only the individual growing cells at metaphase and telophase were counted to get rid of cell clusters. The orientation of the cells during mitosis was determined by the line of nucleus centers or perpendicular line of the equatorial plate. The cells in division were visualized by a confocal laser scanning microscope. The angles between the direction of the stress fiber bundles or mitosis direction vs. the reference (the EPM line or the stretch direction) were measured by AutoCAD based free software. Cells were divided into 9 groups (a 10°-wide sector for each group) according to these angles. As per directions, we plotted the distribution of the cells in a given orientation during mitosis on a polar coordinate chart [34].

### Light and Fluorescence Imaging

Brightfield images were collected using an ECLIPSE TE2000-U microscope (Nikon Corporation Precision Equipment Company, Japan) or Olympus 71X microscope (Olympus Corporation, Japan). Confocal images were collected by a LSM 510 META confocal system (Zeiss, Carl Zeiss MicroImaging, Jena, Germany).

## Supporting Information

Figure S1 Negative control of dual immunofluorescence in liver sections. The negative controls of liver cryosections were incubated with PBS, followed by secondary antibodies including FITC-conjugated goat anti-rat IgG and TRITC-conjugated goat anti-rabbit IgG (Jackson ImmunoResearch Laboratories). Bars  = 50 µm.
(0.28 MB TIF)

Figure S2 WB cells (1×103) were seeded per well of the EPM-coated 96-well plates (5 µg/cm2). Cell numbers were assayed every 24 hours with Cell Counting Kit-8 (Dojindo Laboratories, Japan). The growth curve showed that EPM had a slightly inhibitory effect on the proliferation of WB cells 72 hours after seeding (p<0.05).
(0.03 MB TIF)

Figure S3 The effects of patterned BSA on MO of WB cells. A random distribution of the MO of cells on BSA patterns was observed. (a) Brightfield images of WB cells on micropatterned BSA. (b) Statistical charts. Cell number, n = 55. Bars  = 100 µm.
(0.14 MB TIF)

Figure S4 The relative orientation of SFO and MO of WB cells in a static-uniaxialstretch system. (A) A schematic of the uniaxial stretch device. A rectangular silicone strip (0.6 cm in width, f) was placed on the silicone membrane (d) which sealed the culture well. A uniaxial strain was produced in the strip by the displacement of the indenter (b). (B) The strain in the silicone strip. The longitudinal strain was detected as 4% when the indenter was turned one cycle, while the lateral strain was negligible. (C) Images of the individual cells undergoing uniaxial stretch. 8% strain was exerted on the adherent cells for 24 hours. Cells in division were visualized by F-actin and nuclear staining. The arrowhead indicates strain direction. (D) Cells were plotted in polar coordinate charts according to the angles between the SFO/MO and the stress direction. The uniaxial stretch-induced SFO of cells (cell number: n = 134) or MO (cell number: n = 148) was mostly in the stretch direction. The division of La-A-treated cells appeared to be randomly oriented (cell number: n = 164). Bars, brightfield images: 100 µm, fluorescence images: 50 µm.
(1.42 MB TIF)

Figure S5 Confocal images of the WB cells cultured on fibronectin. Fibronectin did not induce duct formation of WB cells in vitro. Fibronectin-treated cells were stained with rhodamine-labelled phalloidin (red), and anti-tubulin antibody (green) 2–3 days after seeding. Bars  = 100 µm.
(0.25 MB TIF)

Table S1 The relative orientation between SFO and MO in static-uniaxial-stretch system.
(0.03 MB DOC)

## References

[1] Lubarsky B, Krasnow MA (2003). Tube morphogenesis: making and shaping biological tubes.. Cell.

[2] Lemaigre FP (2003). Development of the biliary tract.. Mech Dev.

[3] Yanai M, Tatsumi N, Hasunuma N, Katsu K, Endo F (2008). FGF signaling segregates biliary cell-lineage from chick hepatoblasts cooperatively with BMP4 and ECM components in vitro.. Dev Dyn.

[4] Radisky DC, Hirai Y, Bissell MJ (2003). Delivering the message: epimorphin and mammary epithelial morphogenesis.. Trends Cell Biol.

[5] Radisky DC, Stallings-Mann M, Hirai Y, Bissell MJ (2009). Single proteins might have dual but related functions in intracellular and extracellular microenvironments.. Nat Rev Mol Cell Biol.

[6] Hirose M, Watanabe S, Oide H, Kitamura T, Miyazaki A (1996). A new function of Ito cells in liver morphogenesis: evidence using a novel morphogenic protein, epimorphin, in vitro.. Biochem Biophys Res Commun.

[7] Yoshino R, Miura K, Segawa D, Hirai Y, Goto T (2006). Epimorphin expression and stellate cell status in mouse liver injury.. Hepatology Research.

[8] Segawa D, Miura K, Goto T, Ohshima S, Mikami K (2005). Distribution and isoforms of epimorphin in carbon tetrachloride-induced acute liver injury in mice.. J Gastrol Hepatol.

[9] Watanabe S, Hirose M, Wang XE, Ikejima K, Oide H (1998). A novel hepatic stellate (Ito) cell-derived protein, epimorphin, plays a key role in the late stages of liver regeneration.. Biochem Biophys Res Commun.

[10] Miura K, Yoshino R, Hirai Y, Goto T, Ohshima S (2007). Epimorphin, a morphogenic protein, induces proteases in rodent hepatocytes through NF-kappaB.. J Hepatol.

[11] Miura K, Nagai H, Ueno Y, Goto T, Mikami K (2003). Epimorphin is involved in differentiation of rat hepatic stem-like cells through cell–cell contact.. Biochem Biophys Res Commun.

[12] Wang Y, Wang L, Iordanov H, Swietlicki EA, Zheng Q (2006). Epimorphin(−/−) mice have increased intestinal growth, decreased susceptibility to dextran sodium sulfate colitis, and impaired spermatogenesis.. J Clin Invest.

[13] Hirai Y, Lochter A, Galosy S, Koshida S, Niwa S (1998). Epimorphin functions as a key morphoregulator for mammary epithelial cells.. J Cell Biol.

[14] Coleman WB, Grisham JW, Strain AJ, Diehl AM (1998). Epithelial stem-like cells of the rodent liver.. Liver Growth and Repair: From Basic Science to Clinical Practic.

[15] Coleman WB, Wennerberg AE, Smith GJ, Grisham JW (1993). Regulation of the differentiation of diploid and some aneuploid rat liver epithelial (stemlike) cells by the hepatic microenvironment.. Am J Pathol.

[16] Fan J, Shen H, Dai Q, Minuk GY, Burzynski FJ (2009). Bone morphogenetic protein-4 induced rat hepatic progenitor cell (WB-F344 cell) differentiation toward hepatocyte lineage.. J Cell Physiol.

[17] Chapman L, Coleman WB, Hixson DC (1999). Integration, survival, and differentiation of liver epithelial cells in hepatic and pancreatic ducts.. FASEB J.

[18] Couchie D, Holic N, Chobert MN, Corlu A, Laperche Y (2002). In vitro differentiation of WB-F344 rat liver epithelial cells into the biliary lineage.. Differentiation.

[19] Yao H, Jia Y, Zhou J, Wang J, Li Y (2009). RhoA promotes differentiation of WB-F344 cells into the biliary lineage.. Differentiation.

[20] Hirai Y (1994). Sodium-dodecyl-sulfate-resistant complex formation of epimorphin monomers and interaction of the 150-kDa complex with the cell surface.. Eur J Biochem.

[21] Zhang L, Ishikawa O, Takeuchi Y, Miyachi Y (1998). Immunohistochemical distribution of epimorphin in human and mouse tissues.. Histochem J.

[22] Sicklick JK, Choi SS, Bustamante M, McCall SJ, Pérez EH (2006). Evidence for epithelial-mesenchymal transitions in adult liver cells.. Am J Physiol Gastrointest Liver Physiol.

[23] Oka Y, Hirai Y (1996). Inductive influences of epimorphin on endothelial cells in vitro.. Exp Cell Res.

[24] Grisham JW, Coleman WB, Smith GJ (1993). Isolation, culture, and transplantation of rat hepatocytic precursor (stem-like) cells.. Proc Soc Exp Biol Med.

[25] Germain L, Noël M, Gourdeau H, Marceau N (1988). Promotion of growth and differentiation of rat ductular oval cells in primary culture.. Cancer Res.

[26] Zaret K (1999). Developmental competence of the gut endoderm: genetic potentiation by GATA and HNF3/fork head proteins.. Dev Biol.

[27] Coffinier C, Gresh L, Fiette L, Tronche F, Schütz G (2002). Bile system morphogenesis defects and liver dysfunction upon targeted deletion of HNF1β.. Development.

[28] Tan Y, Yoshida Y, Hughes DE, Costa RH (2006). Increased expression of hepatocyte nuclear factor 6 stimulates hepatocyte proliferation during mouse liver regeneration.. Gastroenterology.

[29] Tang DQ, Lu S, Sun YP, Rodrigues E, Chou W (2006). Reprogramming liver-stem WB cells into functional insulin-producing cells by persistent expression of Pdx1- and Pdx1-VP16 mediated by lentiviral vectors.. Lab Invest.

[30] Chen CS, Mrksich M, Huang S, Whitesides GM, Ingber DE (1997). Geometric control of cell life and death.. Science.

[31] Singhvi R, Kumar A, Lopez GP, Stephanopoulos GN, Wang DI (1994). Engineering cell shape and function.. Science.

[32] Hertwig O (1884). Das Problem der Befruchtung une der Isotropie des Eies, eine Theory der Vererbung. Jenaische Zeitschrift..

[33] Théry M, Bornens M (2006). Cell shape and cell division.. Curr Opin Cell Biol.

[34] Théry M, Racine V, Pépin A, Piel M, Chen Y (2005). The extracellular matrix guides the orientation of the cell division axis.. Nat Cell Biol.

[35] Toyoshima F, Nishida E (2007). Integrin-mediated adhesion orients the spindle parallel to the substratum in an EB1- and myosin X-dependent manner.. EMBO J.

[36] Toyoshima F, Nishida E (2007). Spindle orientation in animal cell mitosis: roles of integrin in the control of spindle axis.. J Cell Physiol.

[37] Fernández-Miñán A, Martín-Bermudo MD, González-Reyes A (2007). Integrin Signaling Regulates Spindle Orientation in Drosophila to Preserve the Follicular-Epithelium Monolayer.. Curr Biol.

[38] Woolner S, O'Brien LL, Wiese C, Bement WM (2008). Myosin-10 and actin filaments are essential for mitotic spindle function.. J Cell Biol.

[39] Hynes RO (2002). Integrins: bidirectional, allosteric signaling machines.. Cell.

[40] Bökel C, Brown NH (2002). Integrins in development: moving on, responding to, and sticking to the extracellular matrix.. Dev Cell.

[41] Eastwood M, Mudera VC, McGrouther DA, Brown RA (1998). Effect of precise mechanical loading on fibroblast populated collagen lattices: morphological changes.. Cell Motil Cytoskeleton.

[42] Hirai Y, Nelson CM, Yamazaki K, Takebe K, Przybylo J (2007). Non-classical export of epimorphin and its adhesion to αv-integrin in regulation of epithelial morphogenesis.. J Cell Sci.

[43] Simian M, Hirai Y, Navre M, Werb Z, Lochter A (2001). The interplay of matrix metalloproteinases, morphogens and growth factors is necessary for branching of mammary epithelial cells.. Development.

[44] Streuli CH (2009). Integrins and cell-fate determination.. J Cell Sci.

[45] LaFlamme SE, Nieves B, Colello D, Reverte CG (2008). Integrins as regulators of the mitotic machinery.. Curr Opin Cell Biol.

[46] Tsao MS, Smith JD, Nelson KG, Grisham JW (1984). A diploid epithelial cell line from normal adult rat liver with phenotypic properties of “oval” cells.. Exp Cell Res.

[47] Duncan AW, Dorrell C, Grompe M (2009). Stem cells and liver regeneration.. Gastroenterology.

[48] Hirai Y, Radisky D, Boudreau R, Simian M, Stevens ME (2001). Epimorphin mediates mammary luminal morphogenesis through control of C/EBPβ.. J Cell Biol.

[49] Hirai Y, Takebe K, Takashina M, Kobayashi S, Takeichi M (1992). Epimorphin: a mesenchymal protein essential for epithelial morphogenesis.. Cell.

[50] Hammarback JA, Letourneau PC (1986). Neurite extension across regions of low cell-substratum adhesivity: implications for the guidepost hypothesis of axonal pathfinding.. Dev Biol.

[51] Matsuda T, Inoue K, Sugawara T (1990). Development of micropatterning technology for cultured cells.. ASAIO Transactions.

[52] Clark P, Britland S, Connolly P (1993). Growth cone guidance and neuron morphology on micropatterned laminin.. J Cell Sci.

[53] Lom B, Healy KE, Hockberger PE (1993). A versatile technique for patterning biomolecules onto glass coverslips.. J Neurosci Methods.

[54] Ranieri JP, Bellamkonda R, Jacob J, Vargo TG, Gardella JA (1993). Selective neuronal cell attachment to a covalently patterned monoamine on fluorinated ethylene propylene films.. J Biomed Mater Res.

[55] Lee AA, Delhaas T, Waldman LK, MacKenna DA, Villarreal FJ (1996). An equibiaxial strain system for cultured cells.. Am J Physiol.

