# Supplementary figures and images for: Epimorphin Regulates Bile Duct Formation via Effects on Mitosis Orientation in Rat Liver Epithelial Stem-Like Cells
Source: PLoS One. 2010 Mar 17;5(3):e9732. doi: 10.1371/journal.pone.0009732 (PMC2840022; doi:10.1371/journal.pone.0009732)

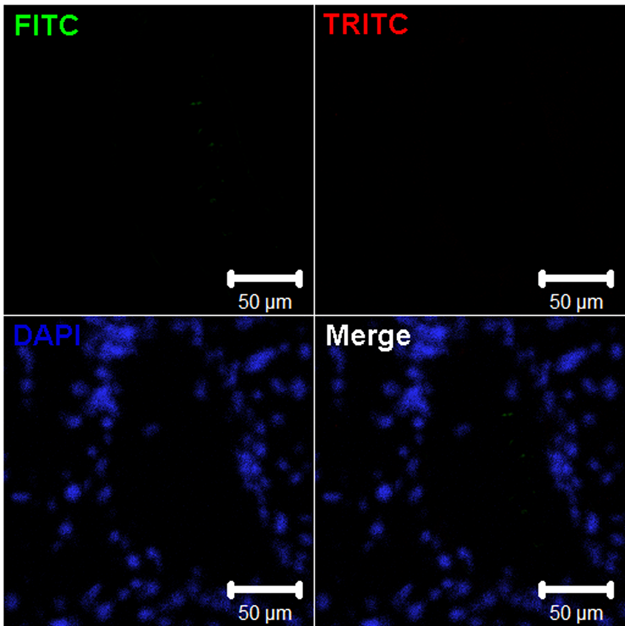

Supplement: Figure S1 — Negative control of dual immunofluorescence in liver sections. The negative controls of liver cryosections were incubated with PBS, followed by secondary antibodies including FITC-conjugated goat anti-rat IgG and TRITC-conjugated goat anti-rabbit IgG (Jackson ImmunoResearch Laboratories). Bars = 50 µm. (0.28 MB TIF) [file pone.0009732.s001.tif]

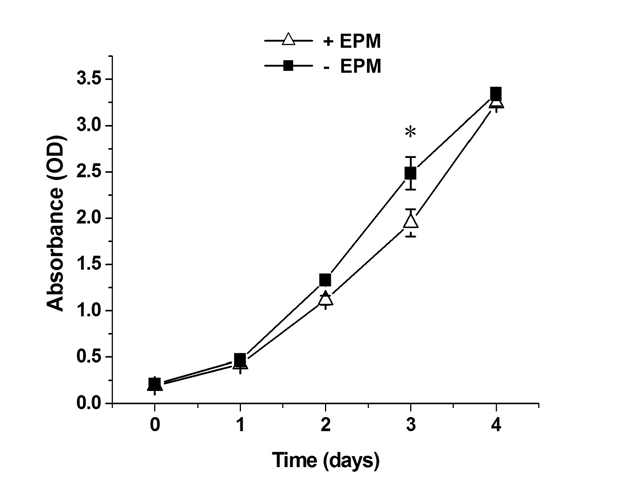

Supplement: Figure S2 — WB cells (1×10^3^) were seeded per well of the EPM-coated 96-well plates (5 µg/cm2). Cell numbers were assayed every 24 hours with Cell Counting Kit-8 (Dojindo Laboratories, Japan). The growth curve showed that EPM had a slightly inhibitory effect on the proliferation of WB cells 72 hours after seeding (p<0.05). (0.03 MB TIF) [file pone.0009732.s002.tif]

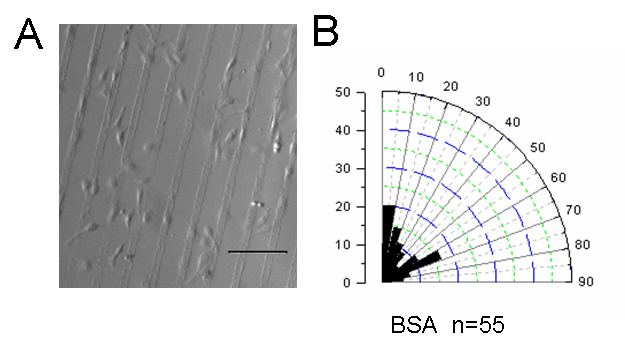

Supplement: Figure S3 — The effects of patterned BSA on MO of WB cells. A random distribution of the MO of cells on BSA patterns was observed. (a) Brightfield images of WB cells on micropatterned BSA. (b) Statistical charts. Cell number, n = 55. Bars = 100 µm. (0.14 MB TIF) [file pone.0009732.s003.tif]

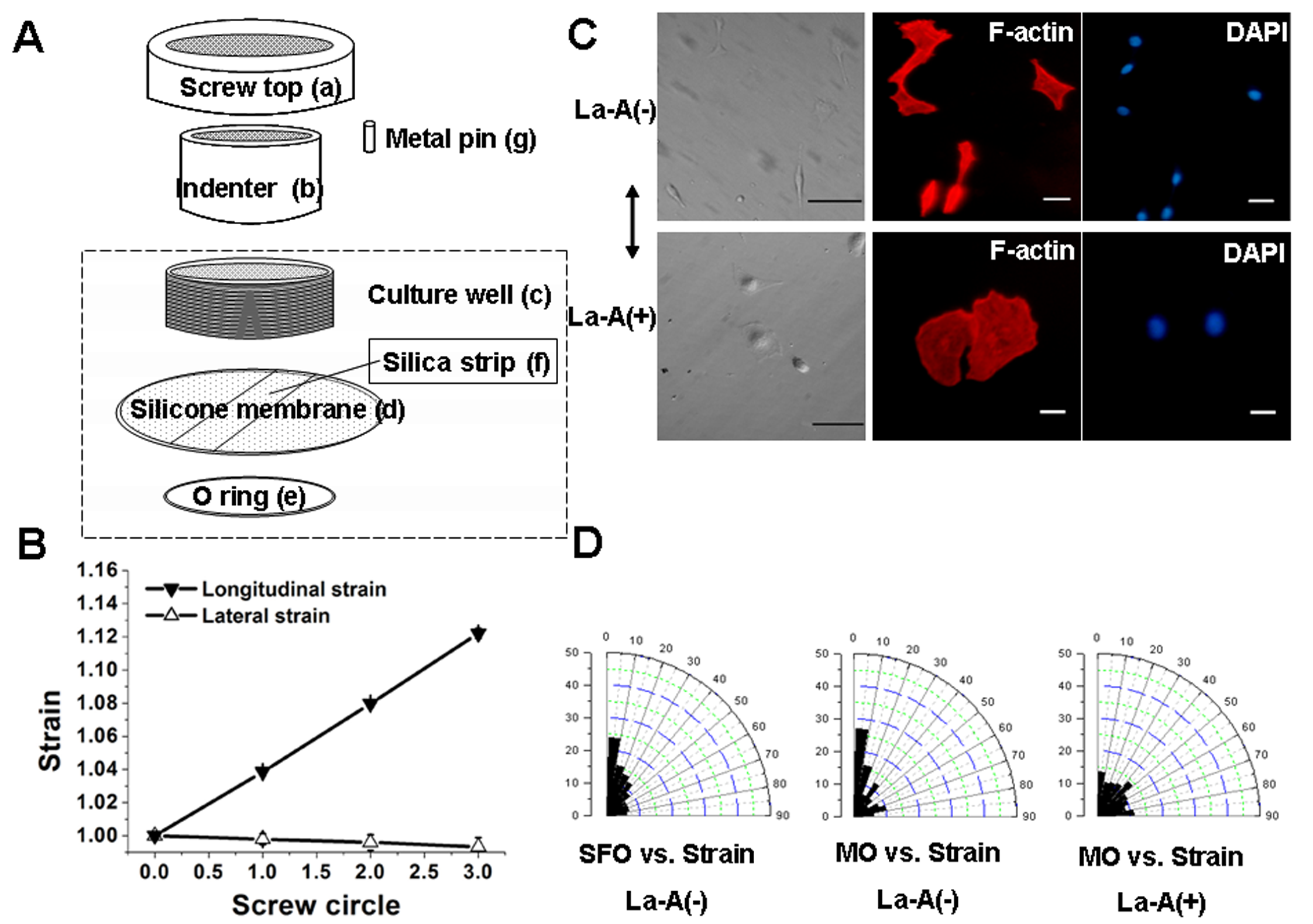

Supplement: Figure S4 — The relative orientation of SFO and MO of WB cells in a static-uniaxialstretch system. (A) A schematic of the uniaxial stretch device. A rectangular silicone strip (0.6 cm in width, f) was placed on the silicone membrane (d) which sealed the culture well. A uniaxial strain was produced in the strip by the displacement of the indenter (b). (B) The strain in the silicone strip. The longitudinal strain was detected as 4% when the indenter was turned one cycle, while the lateral strain was negligible. (C) Images of the individual cells undergoing uniaxial stretch. 8% strain was exerted on the adherent cells for 24 hours. Cells in division were visualized by F-actin and nuclear staining. The arrowhead indicates strain direction. (D) Cells were plotted in polar coordinate charts according to the angles between the SFO/MO and the stress direction. The uniaxial stretch-induced SFO of cells (cell number: n = 134) or MO (cell number: n = 148) was mostly in the stretch direction. The division of La-A-treated cells appeared to be randomly oriented (cell number: n = 164). Bars, brightfield images: 100 µm, fluorescence images: 50 µm. (1.42 MB TIF) [file pone.0009732.s004.tif]

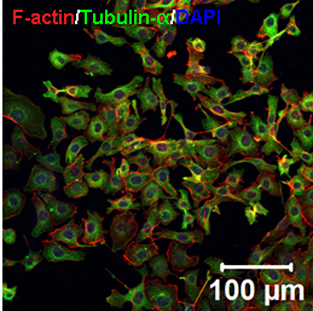

Supplement: Figure S5 — Confocal images of the WB cells cultured on fibronectin. Fibronectin did not induce duct formation of WB cells in vitro. Fibronectin-treated cells were stained with rhodamine-labelled phalloidin (red), and anti-tubulin antibody (green) 2–3 days after seeding. Bars = 100 µm. (0.25 MB TIF) [file pone.0009732.s005.tif]
