# Supplementary material for: Epimorphin Regulates Bile Duct Formation via Effects on Mitosis Orientation in Rat Liver Epithelial Stem-Like Cells
Source: PLoS One. 2010 Mar 17;5(3):e9732. doi: 10.1371/journal.pone.0009732 (PMC2840022; doi:10.1371/journal.pone.0009732)
Supplement: Table S1 — The relative orientation between SFO and MO in static-uniaxial-stretch system. (0.03 MB DOC) [file pone.0009732.s006.doc]

**Table S1. The relative orientation between SFO and MO in static-uniaxial-stretch system***

| ***MO*(strain)**  **SFO(strain)** | 0-30° | 30-60° | 60-90° | **Total** |
| --- | --- | --- | --- | --- |
| 0-30° | 27 | 4 | 0 | 31 |
| 30-60° | 2 | 10 | 1 | 13 |
| 60-90° | 0 | 2 | 4 | 6 |
| **Total** | 29 | 16 | 5 | 50 |

*The cells at metaphase and telophase with visible stress fibers were analyzed. Statistical analysis of the results showed significant correlation between SFO and MO.
